# Distribution Patterns and Environmental Correlates of Invasive Plants Along an Urbanization Gradient in a Medium‐Sized Mountainous City of Southwest China

**DOI:** 10.1002/ece3.74220

**Published:** 2026-10-12

**Authors:** Jiahui Fan, Xiaona Li, Jianying Xiang, Weiwei Li, Zhongjian Deng

**Affiliations:** ^1^ College of Forestry Southwest Forestry University Kunming Yunnan China; ^2^ Yunnan Yuxi Forest Ecosystem National Observation and Research Station Kunming Yunnan China; ^3^ National Plateau Wetlands Research Center, College of Ecology and Environment Southwest Forestry University Kunming Yunnan China; ^4^ Yunnan International Joint Center of Urban Biodiversity Kunming Yunnan China

**Keywords:** alien invasive plants, building density, distribution pattern, Southwest China's mountainous region, urbanization gradient

## Abstract

Against the backdrop of ongoing urban expansion, alien plant invasions are closely linked to the stability of urban ecosystems. However, few studies have documented invasive plant distribution and their statistical associations with landscape variables in mountainous medium‐sized cities, which serve as critical urban–rural transition zones. This study selected Anning City, a representative mountainous medium‐sized city in Yunnan, Southwest China, as the research case. We established 63 standard sampling plots spanning urban, suburban, and exurban zones along an urbanization gradient. Canonical Correspondence Analysis (CCA) and Monte Carlo permutation tests were adopted to quantify statistical associations between six major macro‐environmental variables and invasive plant spatial distribution, and to characterize the distribution features of invasive plants for reference in targeted monitoring work. The main observations are summarized as follows: (1) A total of 66 alien invasive plant species belonging to 24 families and 51 genera were recorded. Asteraceae was the most abundant family, accounting for 25.75% of all taxa; 68.18% of species originated from the Americas, and annual and biennial herbs dominated the community (75.75%), which tend to occur abundantly in frequently disturbed habitats. (2) This local dataset captured a site‐specific trend where invasion abundance declines as urbanization intensity rises: the total number of invasive species increased from urban to suburban and exurban zones, yet significant gradient differentiation only existed in plant abundance rather than species richness. Suburban and exurban plots had similar abundance values (*p* > 0.05), and both presented significantly higher invasive abundance than urban plots (*p* < 0.001). Dense invasive aggregations were frequently detected along transportation corridors and rural land, while forested areas generally corresponded to lower invasive plant coverage. (3) Invasive plant distribution displayed combined statistical associations with multiple environmental variables. Building density presented the strongest correlation with community composition (*p* = 0.004), while urbanization gradient, terrain attributes, and population density also showed significant associations; no meaningful statistical links were detected for road network density and land‐use type. Widespread and common species exhibited significant correlations with macro‐environmental predictors, whereas rare species showed no such associations. Collectively, this paper describes the site‐specific distribution features of invasive plants and their correlated landscape variables in mountainous medium‐sized cities of Southwest China. This work supplements case evidence for invasion ecology research in moderately urbanized mountainous regions and offers empirical references for invasive plant surveillance in comparable subtropical mountainous medium‐sized cities globally.

## Graphical abstract

With accelerated urbanization, alien plant invasions threaten urban ecosystem stability, yet their patterns in medium‐sized mountainous cities remain understudied. We surveyed 63 plots in Anning City, China, finding 66 invasive species and a reverse distribution pattern: invasion richness and abundance increased with decreasing urbanization, with suburban/exurban areas and roads as hotspots, while forests acted as natural barriers. Building density was the core driver of distribution, and we propose targeted management focusing on suburban‐exurban interfaces, common invaders, and native vegetation restoration to guide similar cities globally.

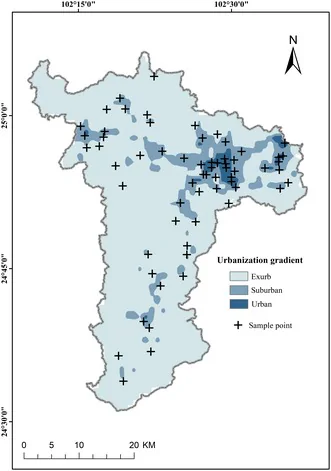

## 1 Introduction

Urbanization is one of the dominant forms of land‐use change worldwide, reshaping habitat structure, microclimate, resource availability, and human disturbance regimes (Wang et al. 2020a, 2020b; Holzmann et al. 2023; Román‐Palacios and Wiens 2020). These changes can influence the establishment and spread of alien plant species, particularly where land conversion, habitat fragmentation, road construction, ornamental planting, and recurrent disturbance increase opportunities for introduction and colonization (Dale and Frank 2018; Alberti et al. 2020; Aronson et al. 2016). Because invasive alien plants can reduce native plant diversity, alter community composition, and impose substantial economic costs (Bradley et al. 2010; Valladares et al. 2015; Diagne et al. 2021), understanding how urbanization filters invasive plant assemblages is important for invasion ecology and urban biodiversity management.

Several theoretical frameworks provide testable expectations for plant invasions along urbanization gradients. The propagule pressure hypothesis predicts higher invasion risk where human activities increase the frequency and quantity of alien plant introductions, such as in densely populated and highly connected urban areas (Lockwood et al. 2005; Colautti et al. 2006; Pyšek et al. 2010). The disturbance‐mediated invasion hypothesis predicts that land‐use change and habitat fragmentation can facilitate invasion by creating establishment opportunities and increasing resource availability (Hobbs and Huenneke 1992; Davis et al. 2000). In contrast, the biotic resistance hypothesis predicts lower invasion success where native communities remain diverse and structurally intact (Levine et al. 2004). The urbanization gradient model further suggests that invasion patterns may vary from urban cores to suburban and exurban areas because propagule pressure, disturbance intensity, habitat availability, and management intensity change simultaneously along this gradient (McDonnell and Hahs 2008; McKinney 2008). Together, these frameworks imply that invasive plant richness may increase toward urban cores or peak in intermediate zones, depending on the balance among introduction pressure, disturbance, habitat availability, and management.

Beyond differences in richness among urban zones, the spatial advance of invasive alien plants within cities is also important for understanding urban invasion dynamics. Urban areas contain multiple pathways through which alien plants can move from introduction points to surrounding habitats, including roadsides, construction sites, residential green spaces, industrial land, river corridors, and disturbed forest edges (von der Lippe and Kowarik 2008; Säumel and Kowarik 2010; Bullock et al. 2018). These pathways may allow invasive species to spread outward from urban cores, inward from peri‐urban or rural source habitats, or laterally along transportation and habitat corridors. Therefore, invasion patterns along urbanization gradients should be interpreted not only as static differences among urban, suburban, and exurban zones, but also as potential stages in the spatial redistribution and spread of invasive species across urban landscapes (McDonnell and Hahs 2008; von der Lippe and Kowarik 2008).

Most empirical studies of urban plant invasion have focused on large metropolitan areas or broad urban–rural comparisons, whereas medium‐sized cities at intermediate stages of urbanization remain less well studied. This scale bias may limit the generality of current invasion theory because medium‐sized cities often combine intensive human activity with relatively continuous semi‐natural habitats. The limitation may be especially important in mountainous cities. Unlike flatland cities, where urbanization gradients are often approximated by distance from the urban core or by impervious surface cover, mountainous cities contain strong topographic heterogeneity, fragmented buildable land, and compressed transitions among built‐up areas, roads, agricultural land, and remnant natural habitats. Mountain systems can impose strong environmental filtering on alien plants, and elevation, terrain, disturbance, and accessibility may jointly shape where non‐native species establish and spread (Becker et al. 2005; Pauchard et al. 2009; Alexander et al. 2011). These conditions may alter the balance among propagule pressure, disturbance, habitat filtering, and biotic resistance, so predictions derived mainly from flatland urban systems may not fully explain invasion patterns in subtropical mountainous cities.

Yunnan Province is a national biodiversity hotspot and is frequently affected by alien plant invasions, hosting a relatively high proportion of China's recorded invasive plant species (Gao and Sun 2021). Anning City, a key city within the Central Yunnan Urban Agglomeration, provides a suitable case for testing whether invasion patterns in a mountainous medium‐sized city follow, modify, or depart from expectations derived from conventional urbanization‐gradient studies. Anning's registered urbanization rate reached 84.92% in 2024 (Anning Municipal People's Government 2025), placing it above China's national average but below highly urbanized megacities. The city forms a continuous urban‐suburban‐exurban gradient, while mountains and hills account for approximately 75% of its total land area. It also experiences multiple disturbance and introduction pathways, including industrial development, transportation expansion, road construction, land‐use transformation, ornamental plant introduction, and natural dispersal (Padayachee et al. 2017). This combination of intermediate urbanization, strong topographic heterogeneity, and multiple introduction pathways makes Anning an appropriate setting for evaluating context‐dependent invasion dynamics in subtropical mountainous cities.

Here, we investigated the spatial distribution of invasive alien plants along the urban‐suburban‐exurban gradient in Anning City. We tested three falsifiable hypotheses. First, if propagule pressure and disturbance dominate invasion patterns, invasive plant richness and abundance should be highest in the urban core; alternatively, if intensive management, high impervious surface cover, and reduced habitat availability limit establishment in the core, richness and abundance should peak in suburban or exurban zones. Based on the mountainous and moderately urbanized setting of Anning, we predicted a non‐linear pattern, with higher invasion levels in intermediate or peripheral zones rather than a simple increase toward the urban core. Second, we hypothesized that invasive plant distributions would be jointly associated with anthropogenic and environmental factors, with building density, population density, and road‐associated disturbance increasing invasion risk, while topographic conditions would mediate where these effects are expressed. Third, we hypothesized that widespread and occasional invasive species would respond differently to the urbanization gradient: widespread invaders should be more strongly associated with human disturbance and connectivity, whereas occasional invaders should show more localized associations with habitat and topographic conditions. By linking these hypotheses to established invasion frameworks, this study evaluates whether mountainous medium‐sized cities require a more context‐specific interpretation of urban invasion dynamics and provides evidence for spatially targeted invasive plant management.

## 2 Materials and Methods

### 2.1 Study Area

Anning City is a core node of the Dianzhong Urban Agglomeration, one of the major urban agglomerations in Southwest China. It is located in central Yunnan Province between 102°08′–102°37′ E and 24°31′–25°06′ N, with a total area of 1301 km^2^. The terrain is mainly mountainous and hilly, accounting for approximately 75% of the total area, with elevations ranging from 1690 to 2617 m. The region has a northern subtropical plateau monsoon climate, with a mean annual temperature of 14.7°C and annual precipitation of approximately 900 mm. The dominant soil types are red soil and yellow soil, accounting for approximately 65% of the area. The vegetation is dominated by subtropical evergreen broad‐leaved forests and mixed coniferous and broad‐leaved forests, with a forest coverage rate of 50.3% (Anning Municipal People's Government, 2025).

As an industrial and transportation hub, Anning contains several pathways for alien plant introduction and spread, including urban greening, land‐use conversion, agricultural activities, roads, and transportation corridors. It also combines an intensive built environment with residual natural habitats. These characteristics make Anning City an appropriate study area for examining invasive plant distributions across an urban‐suburban‐exurban gradient in a mountainous medium‐sized city.

### 2.2 Research Methods

#### 2.2.1 Environmental Variable Screening

Based on the topographic characteristics of subtropical mountainous cities, human disturbance patterns, and existing invasion ecology frameworks, six environmental factors were selected to represent habitat conditions and urbanization‐related disturbance intensity: urbanization gradient (F1), landform (F2), road network density (F3), building density (F4), population density (F5), and land‐use type (F6). The urbanization gradient (F1) was used as the key analytical factor and was constructed through multi‐source spatial data integration, standardization, weighting, and spatial classification (Table 1).

**TABLE 1:** Quantitative standard of environmental factors.

Environmental factor	Quantification standard
F_1_:Urbanization gradient	1 = Urban sample, 2 = Suburban sample, 3 = Exurb sample
F_2_:Landform	1 = Mountains (relative relief ≥ 200 m; Slope ≥ 15°), 2 = Hilly upland (Relative relief 30–200 m; Slope 2°–15°), 3 = Hilly land (Relative relief 30–200 m; Slope < 2°), 4 = gentle slope plain (Relative relief < 30 m; Slope 2°–7°), 5 = Plain (Relative relief < 30 m; Slope < 2°)
F_3_:Road network density	1 = Seldom (0–0.65 km/km^2^), 2 = Path (0.65–1.89 km/km^2^), 3 = Town road (1.89–3.49 km/km^2^), 4 = Suburban main road (3.49–5.56 km/km^2^), 5 = Pivot hub of communications (5.56–9.26 km/km^2^)
F_4_:Building density	1 = Open Land (0%–3.03%), 2 = Scattered Houses (3.03%–9.76%), 3 = Residential Area (9.76%–20.52%), 4 = Mixed‐Use Zone (20.52%–36.54%), 5 = High‐Rise Cluster (36.54%–63.81%)
F_5_:Population density	1 = Sparsely populated (0–238 people/km^2^), 2 = Village (238–941 people/km^2^), 3 = Town (941–2779 people/km^2^), 4 = Suburb (2779–6633 people/km^2^), 5 = Prosperous area (6633–11,866 people/km^2^)
F_6_:Land use types	1 = urban green space, 2 = rural land, 3 = agricultural land, 4 = water land, 5 = Traffic/road land, 6 = wasteland/vacant land, 7 = forest land, 8 = commercial and residential land

Climate variables were not included in the final set of environmental variables. Anning is a plateau city in central Yunnan with a limited administrative area and a relatively narrow geographical span. Within this local study extent, vertical climatic differentiation and spatial climatic heterogeneity are weaker than the variation in landform, land use, road density, building density, and population density. Therefore, climate factors were not selected as core explanatory variables in this study.

#### 2.2.2 Spatial Data Acquisition and Preprocessing

This study used a 1 km grid as the basic spatial analysis unit. This resolution was selected based on the regional terrain complexity, the spatial accuracy of available open‐source urban geographic datasets, and the habitat patch scale of urban and suburban plant communities. It was considered suitable for capturing fine‐scale spatial differentiation in urbanization‐related disturbance within a medium‐sized mountainous city.

Multi‐source spatial data were preprocessed in ArcGIS 10.8, including projection unification, clipping, resampling, and standardization, to reduce dimensional differences and spatial mismatches among datasets. The main data sources were as follows: 2020 building density data and 2019 road network density data were obtained from Science Data Bank (ScienceDB, https://www.scidb.cn/), and 2023 population density data were obtained from Oak Ridge National Laboratory (ORNL), US Department of Energy (https://www.ornl.gov/). These datasets were used to represent artificial construction intensity, traffic accessibility, and population agglomeration intensity, respectively.

#### 2.2.3 Construction of Urbanization Gradient and Zoning Criteria

Three core urbanization indicators, road network density, building density, and population density, were used to construct the integrated urbanization gradient. Because the original GIS datasets already contained density values for each 1 km grid, no additional density calculation formula was required. Instead, the original gridded density values were standardized and reclassified to ensure comparability among indicators with different units and value ranges. The Jenks Natural Breaks classification method was used to divide each standardized indicator into five grades according to its natural spatial distribution.

The Analytic Hierarchy Process (AHP) was used to calculate indicator weights. Five specialists in urban ecology and plant invasion ecology were invited to score the relative importance of the three indicators and construct judgment matrices. The consistency ratio (CR) of all matrices was less than 0.1, meeting the accepted consistency threshold for AHP. The final weight coefficients were 0.38 for building density, 0.33 for road network density, and 0.29 for population density.

Weighted overlay analysis was then performed in ArcGIS 10.8 according to the final weights. Focal statistics spatial smoothing was applied to reduce jagged boundary effects and generate a continuous integrated urbanization‐gradient surface. This surface was further reclassified using Jenks Natural Breaks and combined with Anning's urban functional zoning characteristics to divide the study area into three gradient zones: urban, suburban, and exurban areas (Figure 1).

**FIGURE 1:**
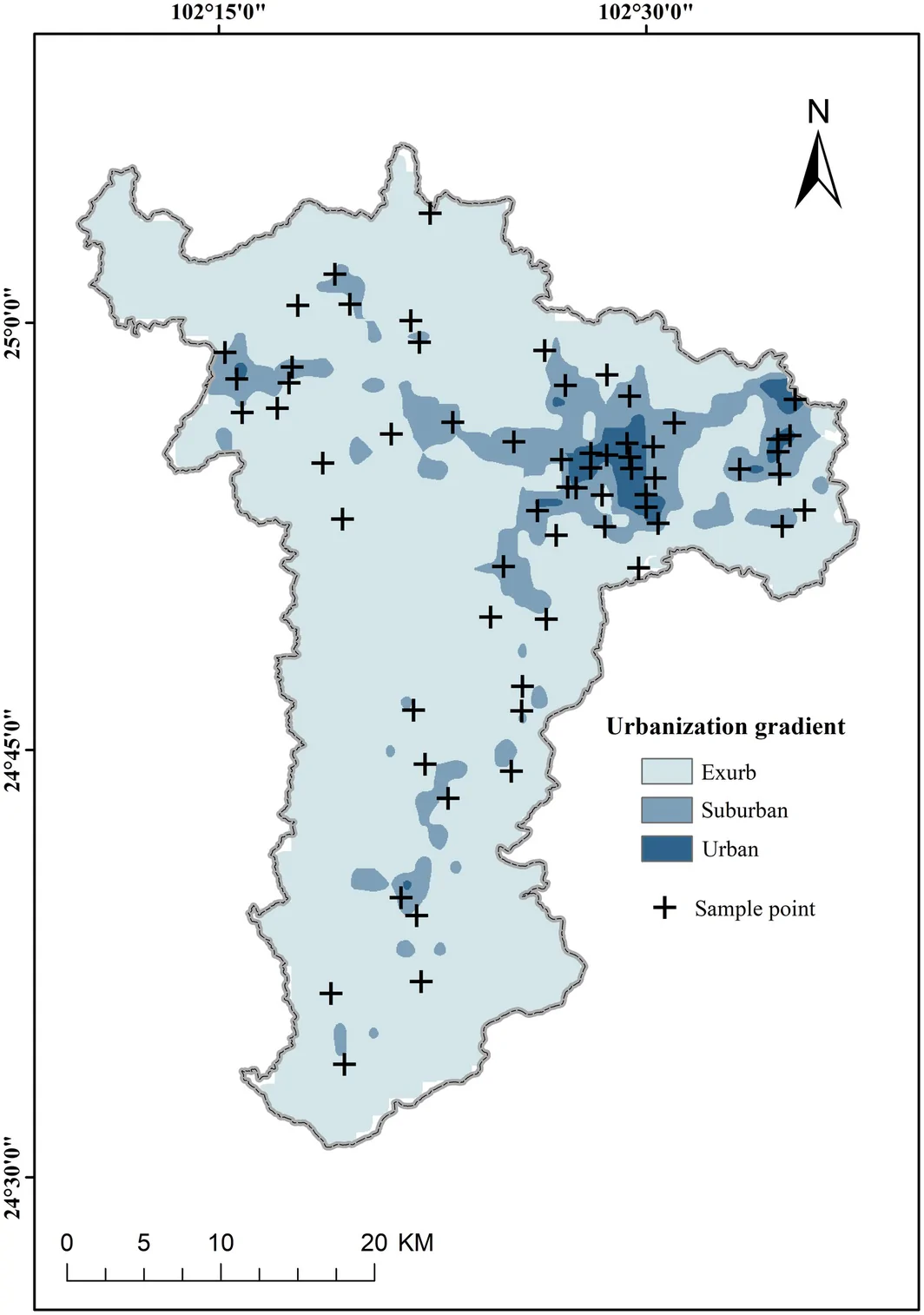
Spatial distribution of urbanization‐gradient zones and sampling units in Anning City, Southwest China. The map shows the classification of urban, suburban, and exurban zones based on the composite urbanization‐gradient index. Black crosses indicate the 63 sampling units used for field surveys.

#### 2.2.4 Field Survey and Sampling Design

A stratified sampling design was adopted based on urbanization‐gradient zones and habitat heterogeneity. Sampling units were arranged to cover high‐disturbance habitats in urban areas, transitional habitats in suburban zones, and mountainous natural or semi‐natural habitats in exurban regions. The number of sampling units was allocated according to the area proportion and habitat complexity of each zone. Specifically, 12 sampling units were established in urban areas, where the area was relatively limited and habitats were more concentrated; 22 sampling units were established in suburban transition zones, where disturbance types were diverse; and 29 sampling units were established in extensive and fragmented mountainous exurban areas. In total, 63 valid sampling units were surveyed.

Sampling locations were selected to cover the main habitat types and disturbance conditions within each gradient zone. Final locations were adjusted after field reconnaissance to ensure accessibility and safety while maintaining coverage of the environmental gradients. The minimum straight‐line distance between sampling units was kept above 500 m to reduce potential spatial autocorrelation.

Field surveys were conducted using reconnaissance surveys combined with transect methods. For dry farmland, irrigated land, orchards, and similar habitats, diagonal or Z‐shaped transects were established, with transect lengths generally ranging from 100 to 500 m and widths of 1–5 m depending on vegetation cover and field visibility. For reservoirs, lakes, paddy fields, and other aquatic habitats, three‐line or diagonal transects were used; transect lengths generally ranged from 100 to 500 m and were adjusted according to water‐area size, with transect widths extending 1 to 5 m landward or waterward along the shoreline to record invasive plants in land‐water ecotones. Along traffic routes and water corridors, parallel transects were arranged following the corridor direction, with lengths of 100 to 500 m and widths of 2 to 5 m on one side of the corridor.

Two field surveys were conducted in October 2024 and April 2025 to cover the main growth periods of herbaceous invasive plants and reduce phenological bias. Species richness was defined as the number of invasive plant species recorded in each sampling unit. Species abundance was quantified using an ordinal five‐level visual cover scale: Grade 1, < 5%; Grade 2, 5%–20%; Grade 3, 20%–40%; Grade 4, 40%–60%; and Grade 5, > 60%. During the surveys, species identity, community cover, and habitat environmental attributes were recorded, and photographs and specimens were collected for species verification when needed.

Following existing classification criteria for invasive plant distribution types (Gao et al. 2011) and considering the occurrence frequency of each species across the 63 sampling units, invasive plants were classified into five distribution types: widely distributed species (frequency ≥ 25%), common species (10% ≤ frequency < 25%), scattered species (3% ≤ frequency < 10%, with sporadic occurrence and no clear aggregation), localized species (low‐frequency species concentrated in specific suitable habitats), and occasional species (frequency < 3%). This frequency‐based classification was used to distinguish differences in distribution breadth and habitat association among invasive species.

#### 2.2.5 Data Processing and Statistical Analysis

Venn diagrams were used to visualize shared and unique invasive plant species among different urbanization‐gradient zones and land‐use types. The Kruskal–Wallis *H* test and post hoc Nemenyi multiple comparison test were used to detect significant differences in invasive plant richness and abundance among gradient zones and habitat types.

Before ordination analysis, multicollinearity among the six explanatory variables was assessed using variance inflation factors (VIFs) with the car package in R. All variables had VIF values below 10 (F1 = 3.18, F2 = 1.51, F3 = 2.74, F4 = 1.35, F5 = 2.15, and F6 = 1.18), indicating that severe multicollinearity was not detected. Therefore, all six environmental variables were retained for subsequent analysis.

Canonical Correspondence Analysis (CCA) was performed using the vegan package in R to explore the relationships between environmental factors and invasive plant community composition. For the ordination analysis, we constructed a 63 sampling units × 66 species abundance matrix and a corresponding environmental matrix. In the environmental matrix, the six environmental variables were summarized for each of the 66 invasive plant species by calculating the mean environmental value across all sampling units in which each species occurred. This resulted in a 66 species × 6 environmental variables matrix for assessing species‐level associations with environmental gradients.

All recorded invasive species, including species recorded in only one sampling unit, were retained in the analysis because rare and occasional species were part of the distribution pattern examined in this study. To avoid overinterpreting rare‐species effects, the ordination results were interpreted mainly at the community and environmental‐gradient level, and species were also classified by occurrence frequency into widely distributed, common, scattered, localized, and occasional types.

The significance of CCA axes and explanatory variables was tested using Monte Carlo permutation tests with 999 permutations. The *p*‐values of the ordination axes and explanatory variables are reported in the Results section. Correlation analysis was conducted in SPSS 26.0, and figures were prepared using Origin and Photoshop.

## 3 Results

### 3.1 Composition of Invasive Plants in Anning

The results of the field surveys showed that a total of 66 alien invasive species were recorded in Anning City, belonging to 51 genera and 24 families (Appendix S1), with the species composition showing obvious family and genus concentration characteristics. At the family level, Asteraceae was the absolute dominant group, with 17 species accounting for 25.75% of the total number of invasive plants; followed by Fabaceae, with 7 species accounting for 10.6%; and Solanaceae, with 5 species accounting for 7.57%. The total number of species in these three families accounted for 43.92% of the total number of invasive plants in the study area (Figure 2b). Invasive plants originating from the Americas were the most numerous, reaching 45 species, accounting for 68.18% of the total number of species; followed by species originating from Europe and Africa, while the proportion of species originating from Asia and Oceania was relatively low. In terms of life form, herbaceous plants were the dominant type of invasive plants in the study area, among which 50 species were annual and biennial herbs, accounting for 75.75% of the total number of species; 16 species were perennial herbs, accounting for 24.24%.

**FIGURE 2:**
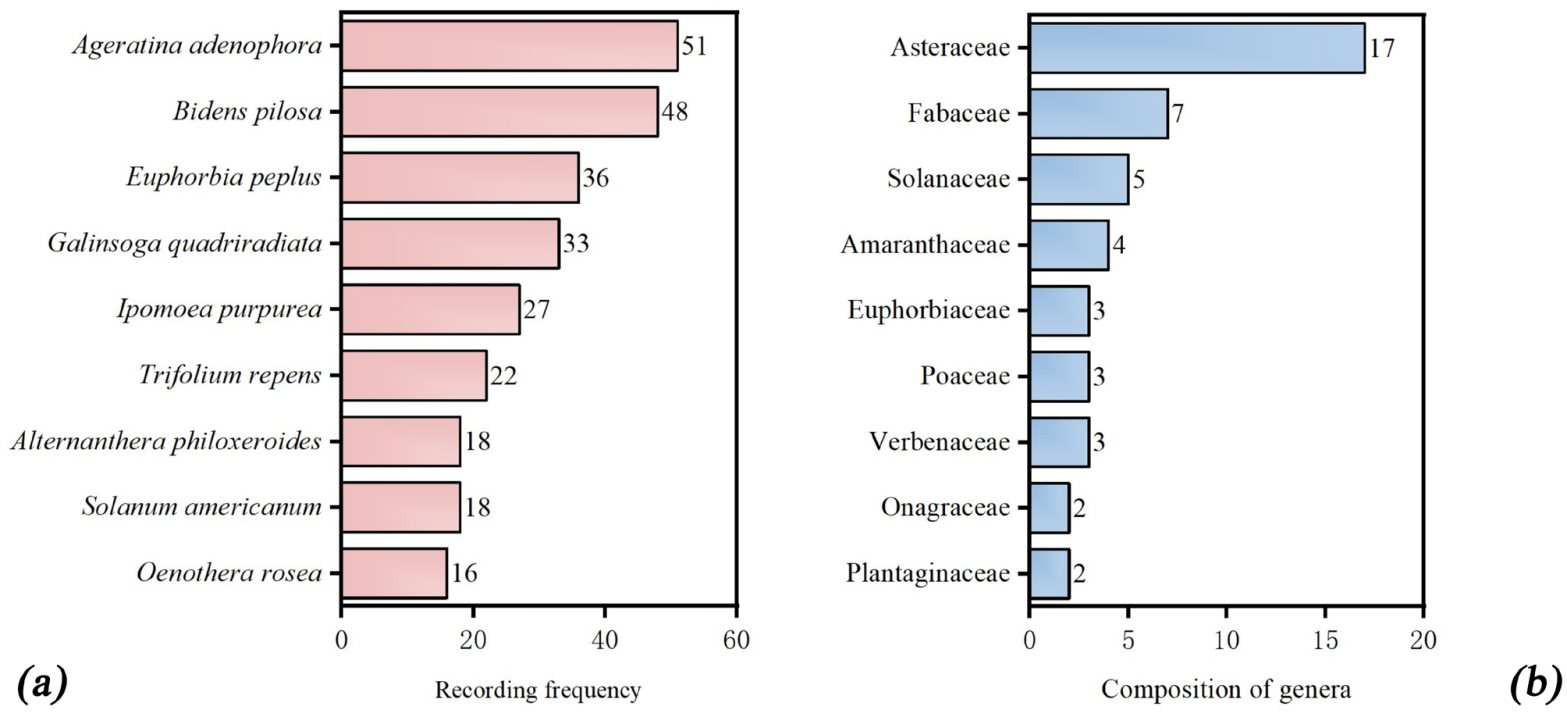
Record frequency (a) and family composition (b) of alien invasive plants.

In terms of the occurrence frequency of invasive plants, there were significant differences in the distribution frequency of invasive plants in the study area. *Ageratina adenophora* had the highest occurrence frequency, reaching 80.95%, followed by *Bidens pilosa* and *Euphorbia peplus* with frequencies of 76.19% and 57.14%, respectively. *Galinsoga quadriradiata* and *Ipomoea purpurea* also had relatively high occurrence frequencies, recorded 33 and 27 times, respectively. Among the 9 widely distributed invasive plants with an occurrence frequency ≥ 25%, 3 belonged to Asteraceae, and the geographical origins of these widely distributed species were mainly the Americas (Figure 2a).

### 3.2 Characteristics of Invasive Plants Under Different Urbanization Gradients

Surveys across urbanization gradients recorded 37, 49, and 54 invasive species in urban, suburban and exurban plots respectively, with species number tending to increase as urbanization weakened. Sampling sizes varied across gradient zones, imposing a minor limitation on this comparison. In terms of species overlap, suburban and exurban plots shared more species relative to urban areas, and 28 invasive species were distributed in all three zones (Figure 4a). Although invasive species richness did not differ significantly along the urbanization gradient (Figure 3b), marked variations were detected in species abundance. No significant abundance difference existed between suburban and exurban plots (*p* > 0.05), yet both zones differed extremely significantly from urban plots (*p* < 0.001, Figure 3a). Specifically, the abundance of suburban plots (*a* = 41.31 ± 3.96) and exurban plots (*a* = 42.24 ± 3.41) was significantly higher than that of urban plots (*a* = 18.91 ± 5.04), and this distribution feature was only reflected in abundance metrics rather than species richness (Table S1 and Table S3).

**FIGURE 3:**
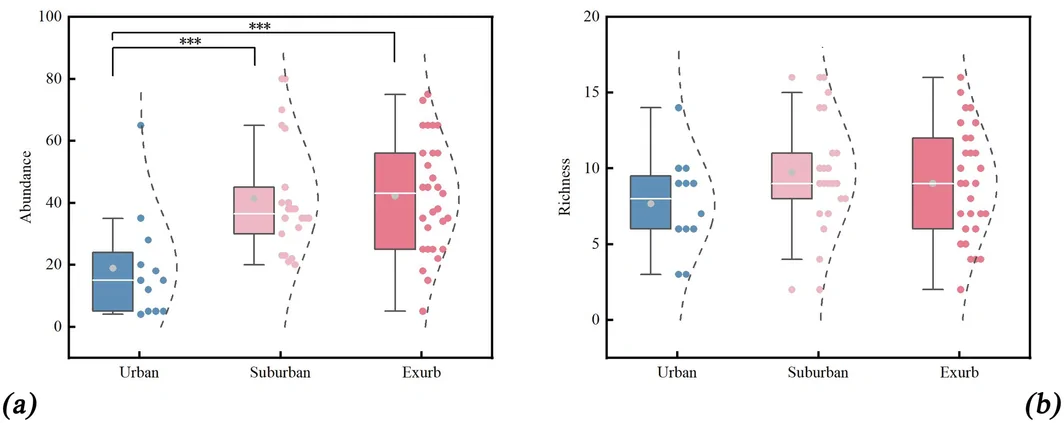
Invasive plant abundance (a) and richness (b) among urbanization gradient. Invasive plant abundance (a) and species richness (b) across the urbanization‐gradient zones in Anning City. Sample sizes were *n* = 12, 22, and 29 for urban, suburban, and exurban zones, respectively. Boxplots are overlaid with individual data points and fitted distribution curves; horizontal lines within boxes indicate medians, and gray points indicate mean values. Differences among zones were tested using the Kruskal–Wallis test followed by post hoc pairwise comparisons. Asterisks indicate significant differences between groups: ***p < 0.001.

### 3.3 Characteristics of Invasive Plants in Different Land Use Types

Survey results of alien invasive plants across different land‐use types revealed significant heterogeneity in both occurrence frequency and species richness among distinct habitats. In terms of occurrence frequency, Traffic/road land supported the highest frequency of invasive plant occurrences, with a cumulative total of 119 records, accounting for 21.29% of the overall occurrence frequency. This was followed by rural land, which had a cumulative occurrence of 85 times, representing 15.21% of the total. In contrast, water areas and forest land exhibited relatively low occurrence frequencies of invasive plants, both below 10%. Among these, forest land had the lowest occurrence frequency, with only 31 records, corresponding to a mere 5.54% of the total (Table 2). *Ipomoea purpurea* , *Ageratina adenophora* , *Galinsoga quadriradiata* , *Bidens pilosa* , *Solanum americanum* , and *Trifolium repens* were the common invasive species across all eight land‐use types. In contrast, *Mirabilis jalapa* and *Senecio vulgaris* occurred exclusively in agricultural land, while *Pontederia crassipes* was only recorded in water areas (Figure 4b). In terms of species richness, Traffic/road land and rural land supported the highest number of invasive plant species, with 39 and 35 species, respectively. Forest land had the lowest species richness of invasive plants, with only 13 species recorded.

**TABLE 2:** Occurrence frequency of alien invasive plants in Anning City.

Land‐use type	Frequency of alien invasive plants	Proportion (%)
Traffic/road land	119	21.29
Rural land	85	15.21
Wasteland/vacant land	78	13.95
Commercial and residential land	72	12.88
Agricultural land	71	12.70
Urban green space	65	11.63
Water land (rivers, canals, lakes, ponds, etc.)	38	6.80
Forest land	31	5.54
Total	559	100

**FIGURE 4:**
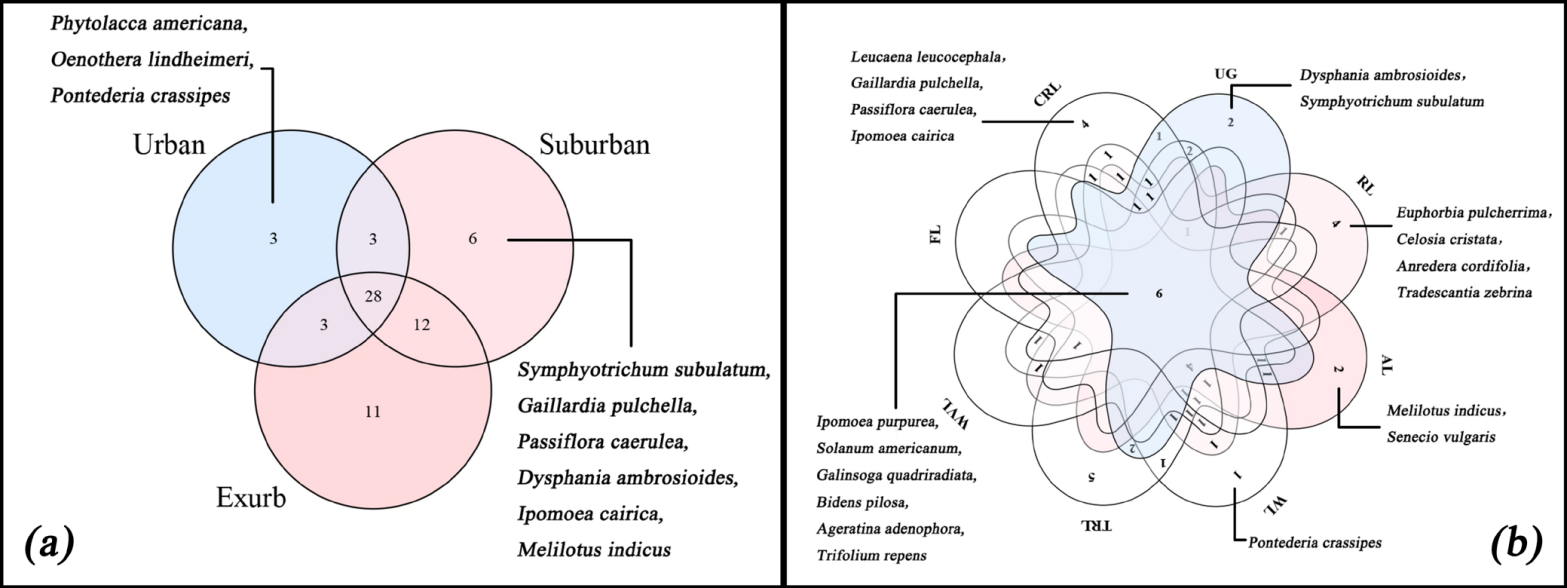
Venn diagram of urbanization gradients (a) and land use types (b). AL, agricultural land; CRL, commercial/residential land; FL, forest land; RL, rural land; TRL, traffic/road land; UG, urban public green space; WL, water area land; WVL, waste/vacant land.

Kruskal–Wallis tests revealed significant differences in both the richness and abundance of invasive plants among different land‐use types (*p* < 0.01). Nemenyi post hoc multiple comparisons indicated that, in terms of species richness, significant differences were observed between Traffic/road land, rural land, commercial/residential land, and woodlands. For abundance, significant differences existed between urban public green spaces, idle/wasteland, and forest land, as well as between commercial/residential land and forest land (Figure 5).

**FIGURE 5:**
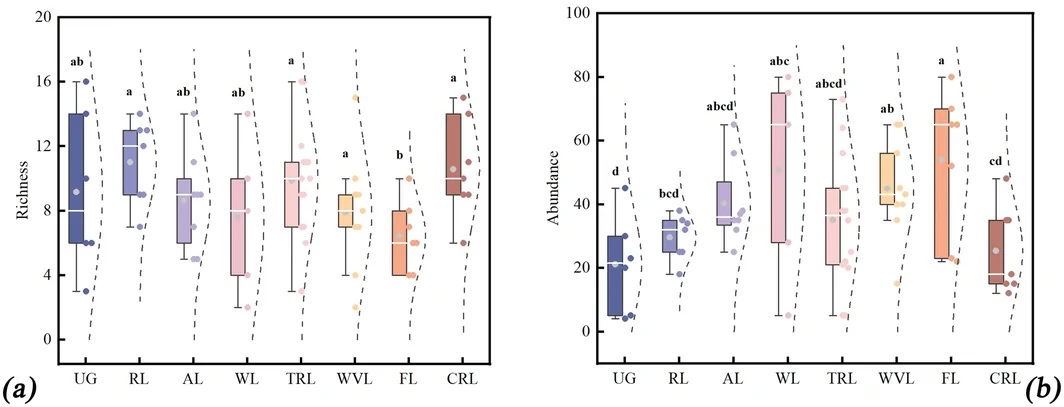
Invasive plant richness (a) and abundance (b) among land use type. Invasive plant species richness (a) and abundance (b) among land‐use types in Anning City. Boxplots are overlaid with individual data points and fitted distribution curves; horizontal lines within boxes indicate medians, and gray points indicate mean values. Differences among land‐use types were tested using the Kruskal–Wallis test followed by post hoc pairwise comparisons. Different lowercase letters indicate significant differences among groups at *p* < 0.05. AL, agricultural land; CRL, commercial/residential land; FL, forest land; RL, rural land; TRL, traffic/road land; UG, urban public green space; WL, water area land; WVL, waste/vacant land.

### 3.4 Effects of Urbanization Gradient Differences on the Distribution of Invasive Plants

#### 3.4.1 Effects of Urbanization Gradient Differences on the Distribution of Invasive Plants With Different Distribution Frequencies

Based on the results of Detrended Correspondence Analysis (DCA), as the length of the ordination axis was greater than 4.0, CCA was chosen to sort and analyze the abundance of invasive plants and the six environmental factors listed in Table 1 under different urbanization gradients in Anning City. The CCA ordination results showed that building density (F4) mainly contributed to CCA axis 1, population density (F5) mainly contributed to CCA axis 2, and topography (F2) had significant contributions to both main axes (Figure 6). The eigenvalues of the first and second axes were 0.274 and 0.239, respectively, and the species‐environment correlation coefficients were 0.820 and 0.768, respectively. The first two axes cumulatively explained 48.4% (Table 3) of the variance in the species‐environment relationship. Ordination results revealed that building density (F4) exhibited the strongest statistical association with invasive plant spatial distributions in Anning City.

**FIGURE 6:**
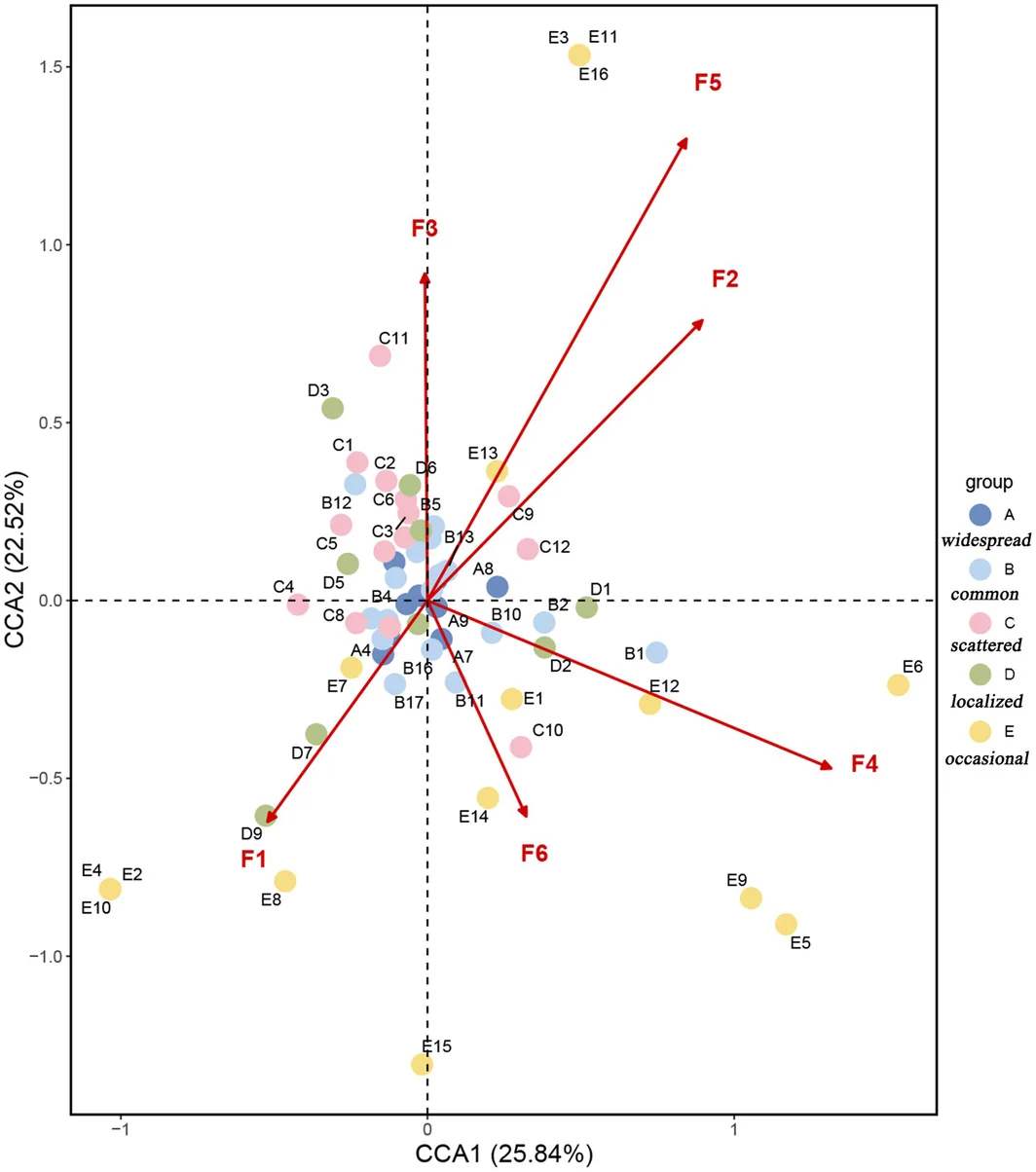
CCA ordination diagram of the relationship between the distribution of invasive plants and environmental factors in Anning City. The arrow in the figure indicates environmental factors, the length of the arrow line indicates the correlation between species distribution and environmental factors, and the quadrant of the arrow indicates the positive and negative correlation between environmental factors and the sorting axis. See Appendix S1 for species and Table 1 for environmental factors F1–F6.

**TABLE 3:** Eigen values and correlations between species axes and environmental axes from CCA ordination.

Item	CCA axes			
	Axis 1	Axis 2	Axis 3	Axis 4
Eigen value	0.274*	0.239	0.184	0.144
Species–environment correlations	0.820	0.768	0.677	0.602
Cumulative percentage variance of species data (%)	2.6	4.9	6.6	8.0
Cumulative percentage variance of species–environment relationship (%)	25.8	48.4	65.8	79.4

* *p* < 0.05.

Monte Carlo permutation testing demonstrated that urbanization gradient (F1), topography (F2), building density (F4), and population density (F5) displayed statistically significant associations with invasive plant distributions (*p* < 0.05, Table 4). Building density (F4) presented an extremely significant statistical correlation with invasive species occurrence (*p* = 0.004) and stood out as the primary correlated variable shaping invasive spatial patterns. Road network density (F3) and land use type (F6) showed no detectable statistical association with invasive plant distributions (*p* > 0.05).

**TABLE 4:** Permutation test results of invasive plants and environmental factors in Anning City.

Environmental factor	*R* ^2^	*p*
F1	0.0175	0.016*
F2	0.0161	0.031*
F3	0.0150	0.120
F4	0.0200	0.004**
F5	0.0193	0.020*
F6	0.0126	0.366

* *p* < 0.05, there is a difference.

** *p* < 0.01, statistically significant difference.

#### 3.4.2 Effects of Urbanization Gradient Differences on the Distribution of Widespread and Common Invasive Plants

Further group analysis was conducted on the five categories of invasive plants (widespread, common, scattered, localized, and occasional). Results of the overall model significance test showed that only the group analysis results of widespread and common invasive plants were statistically significant (*p* < 0.05). The CCA results indicated that the urbanization gradient (F1), building density (F4), and population density (F5) made strong contributions in different directions to CCA Axis 1 (Figure 7). For these two types of species, the eigenvalues between the first and second ordination axes and environmental factors were 0.286 and 0.172, respectively, with species‐environment correlation coefficients of 0.913 and 0.908. The cumulative contribution rate of the species‐environment relationship variance reached 54.8% (Table 5), which was higher than the explanation rate of the overall species. This result indicates that environmental variables display stronger statistical association with the spatial distribution of widespread and common invasive plants.

**FIGURE 7:**
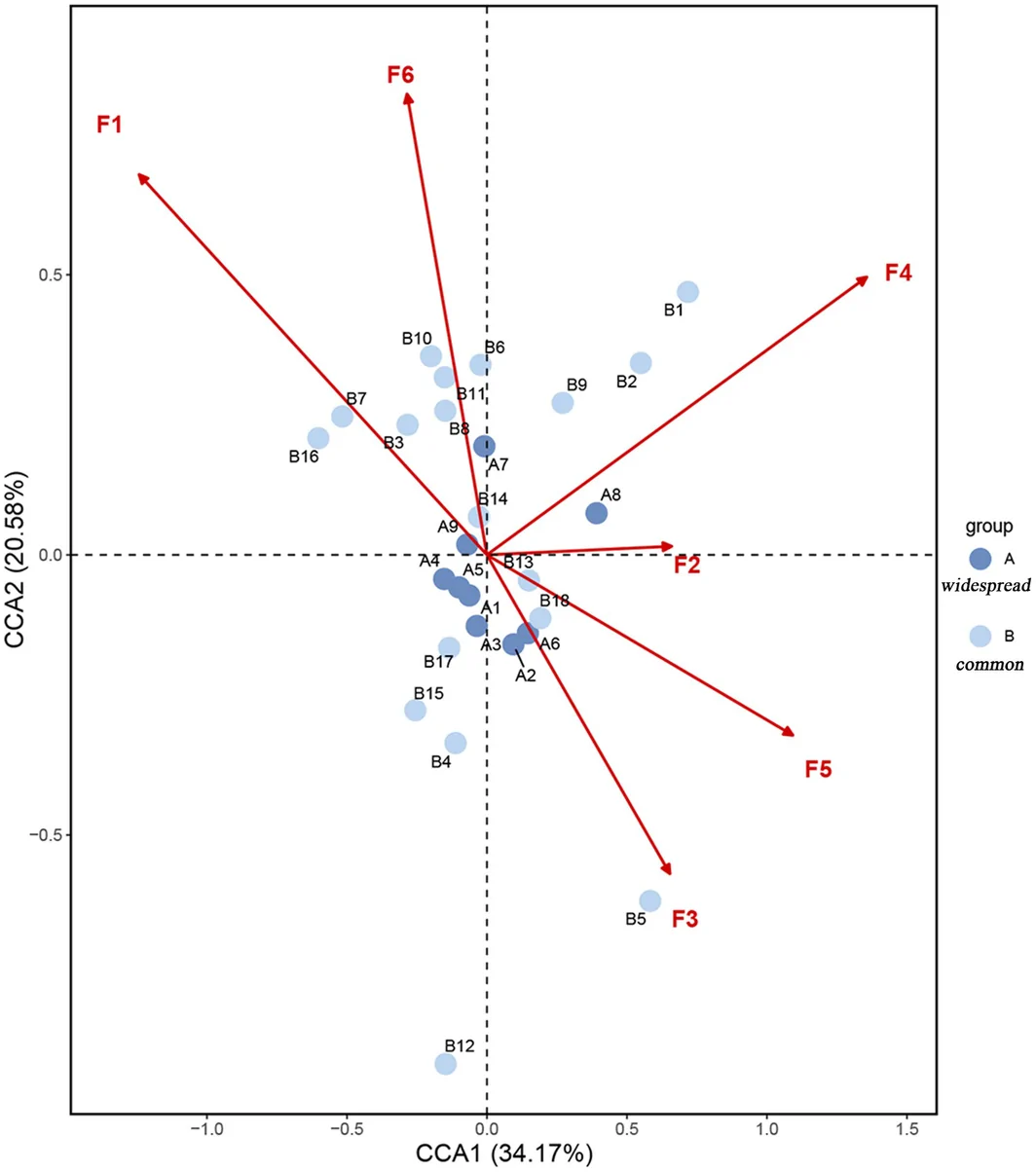
CCA ordination diagram of correlation between disposition and environment factors Eurychoric species; Common species. The arrow in the figure indicates environmental factors, the length of the arrow line indicates the correlation between species distribution and environmental factors, and the quadrant of the arrow indicates the positive and negative correlation between environmental factors and the sorting axis. See Appendix S1 for species and Table 1 for environmental factors F1–F6.

**TABLE 5:** Eigen values and correlations between species and environmental axes from CCA ordination for invading species in Anning.

Item	CCA axes			
	Axis 1	Axis 2	Axis 3	Axis 4
*Eurychoric and common species*				
Eigen value	0.286**	0.172	0.136	0.107
Species–environment correlations	0.913	0.908	0.759	0.862
Cumulative percentage variance of species data (%)	9.1	14.5	18.8	22.2
Cumulative percentage variance of species–environment relationship (%)	34.2	54.8	71.0	83.8

** *p* < 0.01.

Monte Carlo permutation tests (Table 6) reveal significant statistical correlations between the distribution of widespread and common invasive species and two environmental variables: urbanization gradient (F1, *p* = 0.014) and building density (F4, *p* = 0.022, *p* < 0.05).

**TABLE 6:** Permutation test results of environmental factors for Eurychoric and Common invasive plants in Anning City.

Environmental factor	*R* ^2^	*p*
F1	0.0623	0.014*
F2	0.0382	0.303
F3	0.0360	0.423
F4	0.0594	0.022*
F5	0.0425	0.231
F6	0.0379	0.370

* *p* < 0.05, there is a difference.

## 4 Discussion

### 4.1 Invasive Dominance of Asteraceae and Adaptability of Species Originating From the Americas

The family and genus composition of invasive plants in Anning City showed a clear concentration pattern. Asteraceae was the most represented family, with 17 species accounting for 25.75% of all recorded invasive plants. This result is consistent with previous studies showing that Asteraceae is frequently dominant in invasive plant floras, especially in urban and disturbed habitats (Zhang, Du, et al. 2024; Zhang, Zhang, et al. 2024; Zhang et al. 2022; Li et al. 2024). Therefore, the dominance of Asteraceae in Anning does not depart from broader expectations but confirms that the local invasive flora follows a commonly reported taxonomic pattern.

The high representation of Asteraceae may be related to several biological traits. Many species in this family are annual or biennial herbs with short life cycles and high reproductive output. Their seeds often possess accessory structures such as pappus, which facilitate dispersal by wind or water and may enhance colonization in disturbed habitats (Zhang et al. 2022; Wang et al. 2017). In addition, many Asteraceae species have relatively high growth rates and competitive ability in habitats affected by human disturbance. These traits may help explain their frequent occurrence in the mountainous medium‐sized urban landscape of Anning.

Species originating from the Americas accounted for 45 of the recorded invasive species, representing 68.18% of the total. This pattern is consistent with studies from other regions showing that the Americas are a major source area for many alien invasive plants in China (Cui et al. 2022). The high proportion of American‐origin species in Anning may be associated with both ecological adaptability and introduction history. Some of these species are adapted to heterogeneous and disturbed habitats, while others may have been introduced through urban greening or agricultural use and subsequently escaped cultivation. These findings suggest that planting schemes in mountainous cities should give greater attention to native or low‐risk species to reduce potential invasion risks.

### 4.2 Urbanization Gradients, Habitat and Management Correlates of Invasive Plants

Many previous studies have reported positive associations between urbanization intensity and the richness or abundance of invasive plant species (Li et al. 2023; Zhao et al. 2022). In contrast, our field surveys in Anning City showed a different pattern. We recorded 37 invasive species in urban zones, 49 in suburban zones, and 54 in exurban zones, indicating an increasing trend in total species number from urban to exurban areas. However, species richness did not differ significantly among the three gradient zones. Therefore, the reverse pattern observed in this study should be interpreted mainly as an abundance‐based pattern: invasive plant abundance was significantly higher in suburban and exurban plots than in urban plots (*p* < 0.001), whereas suburban and exurban plots did not differ significantly from each other.

This abundance‐based reverse pattern may be associated with Anning's compact urban form, mountainous terrain, and uneven habitat availability across the gradient. The urban core is characterized by high building density, extensive impervious surfaces, and fragmented habitat patches, which may reduce the space available for invasive plant establishment and spread (Li et al. 2023). Urban zones may also receive more frequent green‐space maintenance and weed removal, potentially reducing the persistence of invasive plants in highly managed habitats (Cai and Dong 2017). However, management intensity was not directly measured in this study; therefore, its role should be regarded as a plausible explanation rather than a demonstrated mechanism.

By contrast, suburban and exurban areas contain more extensive natural and semi‐natural habitats, agricultural land, wasteland, idle land, village margins, and road verges. These habitats may provide both dispersal pathways and establishment sites for invasive plants (Zhang et al. 2018; Afonso et al. 2020). This combination of moderate disturbance and greater habitat availability may explain why the pattern observed in Anning differs from that reported in many large metropolitan areas, where urban cores often have stronger propagule pressure, denser transport networks, frequent ornamental introductions, and heterogeneous unmanaged habitats. Thus, the reverse abundance pattern observed here should not be treated as a general ecological rule, but as a context‐dependent pattern associated with Anning's mountainous terrain, compact urban form, peripheral habitat availability, and possible management differences.

### 4.3 Associations Between Land‐Use Types and Invasive Plant Distributions

Across Anning City, invasive plant occurrence metrics varied noticeably among different land‐use categories. Two distinct patterns were observed: high frequencies of invasive species records were detected in areas subject to intensive human disturbance, while natural vegetation sites generally contained fewer invasive individuals and species. Traffic/road land (119 records, 21.29%) and rural land (85 records, 15.21%) emerged as invasion hotspots, with their invasive plant richness reaching 39 and 35 species, respectively, which were significantly higher than those in other land‐use types. The reasons for this phenomenon are as follows: first, the two types of habitats are subject to frequent anthropogenic disturbances. As the core corridor for the spread of invasive plants, roads facilitate the cross‐regional transportation of seeds through vehicles and human activities (Lemke et al. 2021; Deeley and Petrovskaya 2022). Meanwhile, rural land forms continuous habitat disturbances due to agricultural production and residents' daily lives, providing colonization conditions for invasive plants. Second, the habitat types are diverse. The green belts along roads, farmland, and idle land around villages and towns form complex mosaics of habitats, providing diverse ecological niches that adapt to the growth needs of different invasive plants (MacDougall et al. 2009; Paudel et al. 2023). Third, we speculate that limited public awareness of invasive plant risks among rural villagers may lead to unregulated introduction of alien ornamental species and accelerate invasion spread.

Woodland habitats displayed the lowest invasive occurrence frequency (31 records, 5.54%) and lowest species richness (13 species), indicating intact natural vegetation is negatively correlated with plant invasion risk. This finding is consistent with the conclusions of Urbanowicz et al. (2018) and Xu et al. (2016), who demonstrated that woodlands with high canopy closure can effectively restrict the spread and establishment of exotic plants.

Collectively, these correlations suggest that intact habitats tend to correspond with greater resistance to exotic plant establishment, whereas human‐caused habitat fragmentation coincides with dense aggregations of invasive species. These observed associations provide reference for differentiated invasive plant management: priority control can be arranged for heavily disturbed land parcels with abundant invasive records, and undamaged native woodlands ought to be preserved as natural ecological buffers that limit exotic plant spread.

### 4.4 Environmental Correlates of Invasive Plant Assemblages and Species Differential Responses

Results from CCA and Monte Carlo permutation tests reveal statistical associations between the spatial turnover of invasive plant assemblages across Anning City and multiple environmental variables. Among all six measured predictors, building density (F4, *p* = 0.004) exhibited the strongest correlation with community composition; urbanization gradient (F1, *p* = 0.016), terrain features (F2, *p* = 0.031), and human population density (F5, *p* = 0.020) also displayed significant associations. Road network density (F3) and land‐use type (F6) showed no statistically meaningful links to invasive plant composition (*p* > 0.05).

As an indicator representing human disturbance intensity, building density correlates with invasive plant distributions via two interrelated pathways. First, it directly alters habitat structure—high building density corresponds to a high proportion of impervious surfaces and low habitat continuity, which restricts the survival and spread of invasive plants; Second, it indirectly reflects the intensity of human management. Areas with high building density are mostly urban core areas with high management and maintenance levels, whereas areas with low building density have weak management, which is conducive to the establishment of invasive plants (Zhang, Du, et al. 2024; Zhang, Zhang, et al. 2024). The absence of a significant effect of road network density does not mean that it has no role; instead, it is because the core function of road networks is as a propagation corridor, which only affects the spread process of invasive plants. The successful establishment of plants ultimately depends on the comprehensive effects of factors such as building density and topography in the target habitat (Deeley and Petrovskaya 2022), so the effect of road network density is masked by habitat factors.

Clear differences in correlative links to environmental variables were observed among invasive species with distinct occurrence frequencies. The distributions of widespread and common species exhibited significant statistical associations with urbanization gradient (*p* = 0.014) and building density (*p* = 0.022). The first two CCA axes captured 54.8% of species–environment covariation for this group, a larger proportion compared with the 48.4% calculated for all invasive taxa combined. Conversely, no meaningful statistical associations were detected between scattered, localized, occasional species and the measured environmental predictors. Such group‐level differences in correlation patterns may correspond to interspecific differences in niche breadth. Widespread and common species (e.g., *Ageratina adenophora* , *Bidens pilosa* ) tolerate a wide range of urbanization and building density conditions, so their spatial distributions present close statistical links to landscape‐scale environmental metrics. By comparison, low‐frequency species have narrow niche limits and tend to be associated with site‐specific microhabitat attributes (e.g., soil nutrients, fine‐scale local disturbance) that were not quantified within this study, resulting in non‐significant correlations with the selected macro‐environmental variables.

The notable statistical associations between widespread and common species and macro‐environmental variables indicate a potential covariation between their distribution patterns and urbanization gradients. Ongoing urbanization in Anning City may reshape the spatial configuration of building density and population density, which could further correspond to shifts in the distribution of these adaptable species and the assembly of invasive plant communities. Based solely on these correlative patterns, widespread and common species merit targeted monitoring in mountainous medium‐sized cities of Southwest China, with attention focused on their distribution variability across urbanization gradients.

### 4.5 Research Limitations and Future Research Directions

This study explored invasive plant distributions along urbanization gradients in Anning City, but several limitations should be acknowledged. First, the study was conducted in a single county‐level mountainous city with a specific urban form, limited built‐up area, and relatively intensive management in central urban areas. These local socio‐ecological conditions may strongly shape the observed patterns, so the findings should not be directly generalized to other medium‐sized or mountainous cities without comparative evidence. Sampling effort was also unequal among gradient zones, with 12, 22, and 29 sampling units in urban, suburban, and exurban areas, respectively, which may affect comparisons among gradient classes.

Third, the study was based on two short‐term field surveys and lacked long‐term fixed‐plot monitoring. Management intensity was also inferred from landscape context rather than measured directly; therefore, explanations involving green‐space maintenance, weed removal, or urban management should be regarded as hypotheses requiring further testing. Overall, the reverse abundance pattern observed in Anning should not be regarded as a universal ecological rule, but as a context‐dependent pattern associated with the city's urban form, habitat configuration, and possible management regime.

Future research should include multiple cities with different urbanization levels, direct measurements of management intensity, micro‐environmental monitoring, spatial autocorrelation analysis, and long‐term fixed‐plot surveys to better evaluate the processes underlying invasive plant distribution and spread.

## 5 Conclusion

Using Anning City, a medium‐sized mountainous city in Southwest China, as a case study, this research examined the distribution of alien invasive plants across an urban‐suburban‐exurban gradient and evaluated their associations with selected environmental variables. The results provide case‐based evidence for understanding invasive plant distribution in mountainous medium‐sized cities, while their generality should be tested through comparative studies in other regions.

A total of 66 alien invasive plant species from 24 families and 51 genera were recorded. Asteraceae was the most represented family, and most recorded species were annual or biennial herbs. Species originating from the Americas accounted for a high proportion of the invasive flora, suggesting that both biological traits and introduction pathways may contribute to the current species composition.

Along the urbanization gradient, invasive plant abundance was higher in suburban and exurban zones than in urban zones, whereas species richness showed only an increasing trend and did not differ significantly among gradient zones. Therefore, the observed “reverse” pattern should be interpreted mainly as an abundance‐based pattern specific to Anning's urban configuration and habitat conditions, rather than as a general rule for moderately urbanized regions.

Invasive plant distributions also differed among land‐use types. Traffic/road land and rural land showed higher occurrence frequencies and species richness, whereas forested land contained fewer invasive plant records. These patterns indicate statistical associations between land‐use context and invasive plant occurrence, but do not by themselves demonstrate causal mechanisms of hotspot formation.

Among the measured environmental variables, building density showed the strongest statistical association with invasive plant community composition, while urbanization gradient, landform, and population density were also associated with distribution patterns. Widespread and common species showed clearer associations with macro‐environmental variables than low‐frequency species, suggesting that they may deserve priority attention in monitoring programs. Based on these correlative patterns, suburban‐exurban junctions, road verges, and village‐associated habitats may be important areas for targeted monitoring, while maintaining native vegetation continuity may help reduce invasion risk.

## Author Contributions

**Jiahui Fan:** conceptualization (equal), data curation (equal), formal analysis (lead), funding acquisition (equal), investigation (lead), methodology (equal), project administration (equal), resources (equal), software (equal), supervision (equal), validation (equal), visualization (lead), writing – original draft (lead), writing – review and editing (equal). **Zhongjian Deng:** conceptualization (equal), data curation (equal), formal analysis (equal), funding acquisition (lead), investigation (equal), methodology (equal), project administration (lead), resources (lead), software (equal), supervision (equal), validation (lead), visualization (equal), writing – original draft (equal), writing – review and editing (lead). **Jianying Xiang:** conceptualization (equal), formal analysis (equal), methodology (equal), resources (equal), supervision (equal). **Xiaona Li:** conceptualization (supporting), data curation (equal), project administration (equal), software (lead), validation (equal). **Weiwei Li:** data curation (supporting), formal analysis (equal), resources (equal), software (equal), validation (equal).

## Funding

This work was supported by the Key Research and Development Program of Yunnan Province (Grant No. 202403AP140026).

## Conflicts of Interest

The authors declare no conflicts of interest.

## Supporting information


**Table S1:** Kruskal–Wallis tests for invasive plant richness and abundance.
**Table S2:** Bonferroni‐adjusted pairwise comparisons for abundance among urbanization‐gradient zones.

**Table S3:** Descriptive statistics of invasive plant richness and abundance across urbanization‐gradient zones.

**Appendix S1:** The scientific name, life forms, origin and distribution types of 66 alien invasive plants in Anning City.

## Data Availability

All data required to reproduce the analyses in this study are provided as open supplementary data files, including the field‐survey dataset, environmental‐variable matrix, CCA analysis dataset, and SPSS analysis dataset.
