# Supplementary material for: Distribution Patterns and Environmental Correlates of Invasive Plants Along an Urbanization Gradient in a Medium‐Sized Mountainous City of Southwest China
Source: Ecol Evol. 2026 Oct 12;16(10):e74220. doi: 10.1002/ece3.74220 (PMC13658052; doi:10.1002/ece3.74220)
Supplement: Supplementary file 1 [file ECE3-16-e74220-s003.docx]

Appendix I The scientific name, life forms, origin and distribution types of 66 alien invasive plants in Anning City

| No. | Species | Life form | Original place | Distributional types |
| --- | --- | --- | --- | --- |
| A1 | *Ipomoea purpurea* | Liana | America | widespread |
| A2 | *Alternanthera philoxeroides* | Perennial herb | America | widespread |
| A3 | *Oenothera rosea* | Perennial herb | America | widespread |
| A4 | *Solanum americanum* | Annual herb | America | widespread |
| A5 | *Galinsoga* *quadriradiata* | Annual herb | Mexico(America) | widespread |
| A6 | *Bidens pilosa* | Annual herb | America | widespread |
| A7 | *Ageratina* *adenophora* | Perennial herb | Mexico (America) | widespread |
| A8 | *Trifolium repens* | Perennial herb | Europe, Africa, Asia | widespread |
| A9 | *Euphorbia peplus* | Annual herb | Europe, Africa, Asia | widespread |
| B1 | *Oxalis latifolia* | Perennial herb | America | common |
| B2 | *Oxalis corymbosa* | Perennial herb | America | common |
| B3 | *Bougainvillea spectabilis* | Shrub | America | common |
| B4 | *Veronica persica* | Annual or biennial herb | Europe, Asia | common |
| B5 | *Cardamine flexuosa* | Annual or biennial herb | Europe | common |
| B6 | *Acacia dealbata* | Tree | Australia (Oceania) | common |
| B7 | *Avena fatua* | Annual herb | Europe, Africa, Asia | common |
| B8 | *Bromus catharticus* | Annual or biennial herb | America | common |
| B9 | *Ageratum conyzoides* | Annual herb | America | common |
| B10 | *Bidens alba* | Annual herb | America | common |
| B11 | *Erigeron canadensis* | Annual herb | America | common |
| B12 | *Erigeron sumatrensis* | Annual or biennial herb | America | common |
| B13 | *Crassocephalum* *crepidioides* | Annual herb | Africa | common |
| B14 | *Erigeron bonariensis* | Annual or biennial herb | America | common |
| B15 | *Sonchus oleraceus* | Annual or biennial herb | Europe | common |
| B16 | *Solanum aculeatissimum* | Perennial herb | America | common |
| B17 | *Nicandra physalodes* | Annual herb | America | common |
| B18 | *Cyclospermum* *leptophyllum* | Annual herb | America | common |
| C1 | *Ricinus communis* | Annual herb | Africa | scattered |
| C2 | *Vicia villosa* | Annual or biennial herb | Europe, Asia | scattered |
| C3 | *Trifolium hybridum* | Perennial herb | Europe | scattered |
| C4 | *Malvastrum* *coromandelianum* | Perennial herb | America | scattered |
| C5 | *Crassocephalum rubens* | Annual herb | Africa | scattered |
| C6 | *Sonchus asper* | Annual herb | Europe, the Mediterranean | scattered |
| C7 | *Tagetes erecta* | Annual herb | Mexico(America) | scattered |
| C8 | *Lantana camara* | Shrub | America | scattered |
| C9 | *Parthenocissus* *quinquefolia* | Liana | America | scattered |
| C10 | *Datura stramonium* | Annual herb | America | scattered |
| C11 | *Amaranthus cruentus* | Annual herb | America | scattered |
| C12 | *Veronica arvensis* | Annual herb | Europe, Asia | scattered |
| C13 | *Mirabilis jalapa* | Annual herb | America | scattered |
| C14 | *Opuntia dillenii* | Shrub | America | scattered |
| D1 | *Senecio vulgaris* | Annual herb | Europe, Africa | localized |
| D2 | *Duranta erecta* | Shrub | America | localized |
| D3 | *Verbena bonariensis* | Perennial herb | America | localized |
| D4 | *Cyperus involucratus* | Perennial herb | Africa | localized |
| D5 | *Cosmos bipinnatus* | Annual herb | Mexico (America) | localized |
| D6 | *Tradescantia pallida* | Perennial herb | America | localized |
| D7 | *Senna bicapsularis* | Shrub | America | localized |
| D8 | *Lolium perenne* | Perennial herb | Europe, Africa, Asia | localized |
| D9 | *Eucalyptus globulus* | Tree | Australia (Oceania) | localized |
| E1 | *Euphorbia pulcherrima* | Shrub | America | occasional |
| E2 | *Leucaena leucocephala* | Tree | America | occasional |
| E3 | *Melilotus indicus* | Annual herb | South Asia, Central Asia to Southern Europe | occasional |
| E4 | *Symphyotrichum* *subulatum* | Annual herb | America | occasional |
| E5 | *Gaillardia pulchella* | Annual herb | Europe | occasional |
| E6 | *Oenothera lindheimeri* | Perennial herb | America | occasional |
| E7 | *Anredera cordifolia* | Liana | America | occasional |
| E8 | *Solanum pseudocapsicum* | Shrub | America | occasional |
| E9 | *Passiflora caerulea* | Liana | America | occasional |
| E10 | *Dysphania ambrosioides* | Annual herb | America | occasional |
| E11 | *Celosia cristata* | Annual herb | America, Africa, Asia | occasional |
| E12 | *Ipomoea cairica* | Liana | Africa, Asia | occasional |
| E13 | *Tradescantia zebrina* | Perennial herb | Mexico (America) | occasional |
| E14 | *Pontederia crassipes* | Perennial herb | America | occasional |
| E15 | *Opuntia ficus-indica* | Shrub | America | occasional |
| E16 | *Phytolacca americana* | Perennial herb | America | occasional |
